# High grade persistent ventral curvature after multiple hypospadias surgery: how to correct?

**DOI:** 10.1590/S1677-5538.IBJU.2021.0353

**Published:** 2021-06-20

**Authors:** Antonio Macedo, Sérgio Leite Ottoni, Gilmar Garrone, Marcela Leal da Cruz

**Affiliations:** 1 Universidade Federal de São Paulo São Paulo SP Brasil Universidade Federal de São Paulo - UNIFESP, São Paulo, SP, Brasil; 2 CACAU-NUPEP Departamento de Urologia São Paulo SP Brasil Departamento de Urologia, CACAU-NUPEP, São Paulo, SP, Brasil

## Abstract

**Introduction:**

A challenging situation in proximal hypospadias is the presentation of patients with successful urethroplasty but with persistent or recurrent ventral curvature (VC) after multiple hypospadias repair.

**Materials and Methods:**

We present a 13 year-old boy with 7 previous surgeries (long TIP, Duplay, meatoplasty) to treat hypospadias presenting with 60 degrees of VC, in spite of a well-accepted coronally neomeatus. We degloved the penis and artificial erection clearly appointed corporal disproportion causing curvature. We disconnected urethra from corpora. After excision of remnant fibrotic tissue, there was a residual curvature so a lenghtening corporoplasty with dermal graft from groin was performed. We have adjusted the urethral meatus position into a proximal penile shaft. We used a buccal mucosa graft placed in an inverted U-shape position planning a second stage urethroplasty (1). An indwelling silicone Foley tube was left for one week. The patient was discharged the day after surgery.

**Results:**

The aspect after corporoplasty proved satisfactory curvature correction. Patient had an excellent outcome and is scheduled for a second-stage after 6 months.

**Discussion:**

Snodgrass and Bush (2) reported that on 73 patients with an average of 2.7 operations for proximal shaft to perineal hypospadias; of which, 83% had VC at re-operation averaging 50°. We do believe that some good results with minimal dorsal plicature may recur in adolescence and therefore when these procedures may be considered, they should be performed by classic Nesbit technique (3). Otherwise, the choice for primary ventral lengthening should be taken.

**Conclusion:**

Severe curvature associated with hypospadias should undergo a major procedure at early stage to avoid decompensation after dorsal plicature in adolescence. We had a very satisfactory result, the patient awaits the second stage procedure (Figure-1).

**Figure 1:**
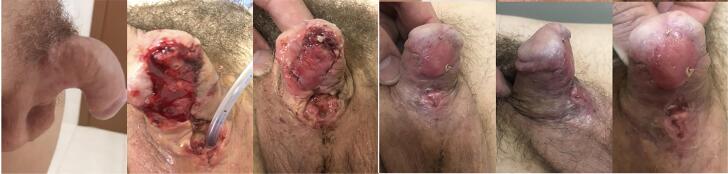
End aspect after complete healing of buccal mucosa. Notice that penis is straight and second stage will be performed after 6 months of interval.

**Figure:** Available at:http://www.intbrazjurol.com.br/video-section/20210353_Cruz_et_al
